# Designing and benchmarking the MULTICOM protein structure prediction system

**DOI:** 10.1186/1472-6807-13-2

**Published:** 2013-02-27

**Authors:** Jilong Li, Xin Deng, Jesse Eickholt, Jianlin Cheng

**Affiliations:** 1 Computer Science Department, University of Missouri, Columbia, MO, USA; 2 Informatics Institute, University of Missouri, Columbia, MO, USA; 3 C. Bond Life Science Center, University of Missouri, Columbia, MO, USA

**Keywords:** Protein structure prediction, Template identification, Template combination, Model generation, Model assessment, Model combination, Model refinement

## Abstract

**Background:**

Predicting protein structure from sequence is one of the most significant and challenging problems in bioinformatics. Numerous bioinformatics techniques and tools have been developed to tackle almost every aspect of protein structure prediction ranging from structural feature prediction, template identification and query-template alignment to structure sampling, model quality assessment, and model refinement. How to synergistically select, integrate and improve the strengths of the complementary techniques at each prediction stage and build a high-performance system is becoming a critical issue for constructing a successful, competitive protein structure predictor.

**Results:**

Over the past several years, we have constructed a standalone protein structure prediction system MULTICOM that combines multiple sources of information and complementary methods at all five stages of the protein structure prediction process including template identification, template combination, model generation, model assessment, and model refinement. The system was blindly tested during the ninth Critical Assessment of Techniques for Protein Structure Prediction (CASP9) in 2010 and yielded very good performance. In addition to studying the overall performance on the CASP9 benchmark, we thoroughly investigated the performance and contributions of each component at each stage of prediction.

**Conclusions:**

Our comprehensive and comparative study not only provides useful and practical insights about how to select, improve, and integrate complementary methods to build a cutting-edge protein structure prediction system but also identifies a few new sources of information that may help improve the design of a protein structure prediction system. Several components used in the MULTICOM system are available at: http://sysbio.rnet.missouri.edu/multicom_toolbox/.

## Background

Predicting protein tertiary structure from sequence is an important and challenging problem in bioinformatics and computational biology [1,2]. Computational protein structure prediction is useful for protein function study, protein design, protein engineering, drug design, and protein evolution analysis [3,4]. It is becoming increasingly important in the post genomic era as millions of new protein sequences are produced by numerous DNA sequencing projects each year, leading to an enlarged knowledge gap between sequences and known experimental structures [5].

During the last few decades, numerous techniques were developed by scientists in multiple disciplines, such as biophysics, computational chemistry, computer science, and bioinformatics, to address different aspects of protein structure prediction. These aspects include secondary structure prediction, solvent accessibility prediction, disordered region prediction, domain boundary prediction, template identification, query-template alignment, template-based model generation, template-free model sampling, loop modeling, model/alignment quality assessment, and model refinement. Although not perfect, many of these methods can produce complementary and useful information to inform the final tertiary structure of a query protein [6,7]. In addition to technological advances, increasing amounts of protein structures have been determined by experimental techniques and provide a rich set of structural data for enhancing protein structure prediction. Thus, it has become an important task to systematically integrate these diverse and complementary methods into a state of the art protein structure prediction system that can mine the enlarging protein sequence and structure databases to accurately and quickly predict the tertiary structure of any query protein [5,8].

In order to integrate diverse protein structure prediction methods and multiple sources of information into one effective system, we have designed an open, five-layer, component-based protein structure prediction pipeline [9] that corresponds to the five major steps of protein structure prediction: template identification, query-template alignment and combination, model generation, model quality assessment, and model refinement. The components in the pipeline are loosely linked through information flow from one layer to next. The input to the pipeline is a query sequence and the output of the previous step is used as input to next step until the final structural models are produced from the pipeline. The interfaces between components are flexible and well designed, so that different methods developed for each step can be easily plugged into the system. Once the system is constructed under the open architecture, the next challenge is to benchmark the system and optimize a large number of parameters of the components. This system then selectively integrates the sequence and structural information produced by these components to generate final protein conformations of good quality. We blindly tested our current implementation of the system, MULTICOM, during the ninth Critical Assessment of Techniques for Protein Structure Prediction (CASP9, http://predictioncenter.org/casp9/) in 2010. The open system delivered very good performance. After the blind prediction phase of CASP9 ended, we systematically analyzed the intermediate data generated by each component in each prediction step and gained a great deal of experience about how to combine and configure components and integrate multiple sources of information in order to build a high-quality protein structure prediction system. In addition to presenting a comprehensive benchmark of the components of the MULTICOM system as tested in CASP9, this work describes a number of new methodological developments incorporated into our system since it was first launched during the CASP8 experiment.

## Methods

### Overview of system architecture, design, and implementation

**Figure 1:**
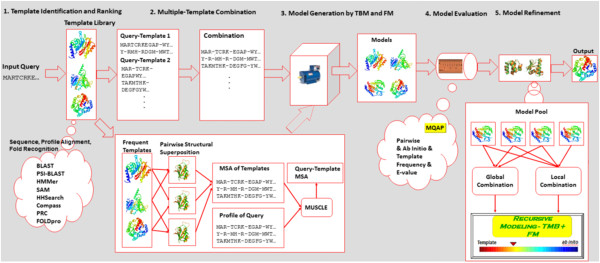
**The five-layer architecture of the MULTICOM protein structure prediction system. **TBM stands for template-based modeling and FM for template-free modeling.

Figure 1 illustrates the architecture of the MULTICOM protein structure prediction system [9]. The system consists of five major layers. The template identification layer accepts an input query sequence and searches it against a non-redundant protein sequence database to construct a query sequence profile. This profile is searched against a template library in order to identify a list of template protein structures that may provide conformation information about the structure of the query. A subset of top ranked templates and their sequence alignments with the query protein if available are fed into the template combination layer, which combines the structurally similar templates and the query into query-template alignments. The query-template alignments may contain more than one template which provides complementary information about the query. Then the systematic combination of multiple templates generates a number of query-template alignments. The query-template alignments and template structures are fed into model generation tools (model generator) to sample conformations for the query. The regions of the query that align with templates are sampled by a template-based model generator (e.g. a comparative modeling tool) and the large (>10 residues) unaligned query regions are sampled by a template-free model generator (e.g. a fragment-assembly tool). The model generators usually produce a number of models, which are then evaluated by the model quality assessment layer. The model quality assessment tools assign a global quality score to each model measuring its overall quality (e.g. overall similarity between the model and the known native structure) and a local quality score to each residue predicting its deviation compared with the native structure. Finally, the models and their predicted quality scores are fed into the last model refinement layer in order to further improve their quality. In this layer, multiple models with similar conformations may be combined (e.g. averaged) and the low-quality regions of some models may be refined by stochastic simulations. At the end, the models with the best predicted qualities are released from the system as the final predictions.

The open architecture of the protein structure prediction system makes it easy to plug in complementary methods as components and integrate multiple sources of information (e.g. template conformations) drawn from the template and sequence library / databases in order to produce high quality models. The subsections below present the implementation of the MULTICOM system emphasizing the new developments occurred since its first version [9] and the components that were thoroughly assessed in this work.

#### Template structure and sequence library

In order to support template-based structural prediction, a template library is constructed from the known experimental structures in the Protein Data Bank [10]. The template library includes template sequence, template structure (i.e. atom coordinates), secondary structure and solvent accessibility derived from the structure by DSSP [11,12], and template sequence profiles. The template profiles are constructed from the multiple sequence alignment of the template sequence and its homologous sequences found by PSI-BLAST [13] when searching the template sequence against the Non-Redundant protein sequence database. The e-value cut off and the number of iterations of PSI-BLAST search range from 0.001 - 0.1 and 3–8, respectively, depending on the difficulty of the query. Different profiles such as HHSearch [14] hidden Markov model, COMPASS [15] profile, PRC [16] hidden Markov model, and PSI-BLAST [13] PSSM are created in order to facilitate a variety of profile-profile alignments. The HHSearch profiles also include the secondary structure information of the template proteins. Two lists of template sequences are created. The big list (LIB-A) essentially includes all the proteins (~60,000) in the PDB before the CASP9 experiment started excluding identical proteins and short proteins (<30 residues). The small list (LIB-B) is a redundancy reduced list filtered at 90% sequence identify, which includes ~20,000 proteins. In order to keep the library updated, the new protein structures released by the PDB are retrieved and incorporated into the library every week. Similarly, the non-redundant sequence database is updated weekly from the NCBI’s web site.

#### Template identification

A query sequence is first searched against the Non-Redundant protein sequence database by PSI-BLAST [13] in order to find its homologous sequences. Query profiles (i.e., PSI-BLAST [13] PSSM, HHSearch [14] HMM, SAM [17] HMM, HMMER [18] HMM, PRC [16] HMM, and COMPASS [15] profile) are constructed from the query and its homologous sequences. Because the template identification is often sensitive to profile content, three kinds of HHSearch profiles are constructed for the query using the small, large, and filtered NR database. One special addition to the HHSearch profiles is that they include the secondary structure of the query protein predicted by either SCRATCH [19] or PSI-PRED [20]. In order to identify a list of template structures potentially relevant to the structure of a query protein, the sequence and its profile are searched against the template sequences and profiles. Specifically, the query sequence is searched against LIB-A using BLAST [13,21] and CSI-BLAST [22]. The query PSSM, SAM, and HMMER profiles are searched against LIB-A by PSI-BLAST, SAM, and HMMER. The query HHSearch, PRC, and COMPASS profiles are searched against the profiles in LIB-B by HHSearch, PRC, and COMPASS. These searches are carried out by multiple threads in parallel. Each search may return a list of templates with e-values below a pre-defined threshold (e.g., 1 for hard targets and 0.001 for easy targets) and the local alignment between the query and templates is also generated. The top ranked template hits ranked by the e-values of the query-template alignments are retained for each method and the query-template alignments from the top hits identified by each method are stored in separate lists for later combination. Furthermore, the system counts the number of times a template was found by each alignment method and generates a consensus list of the top ranked (e.g. top 10) templates ranked solely by the frequency counts. The consensus template selection is a new addition to the MULTICOM system. CSI-BLAST, PRC, HMMER, and SAM are new alignment methods added into the system. It is worth noting that more sequence and profile alignment methods could be easily plugged into this layer, which often improves the performance of the system as multiple search tools often contribute complementary information or reinforce weak signals.

#### Multiple template combination

A template structure directly suggests a conformation that is supposed to be near the native conformation of the query protein being searched. This drastically reduces the search space. Multiple structurally similar templates may provide an ensemble of conformations that better confine the native structure of the query protein [23]. The multiple template combination layer is designed to integrate the structural information from multiple templates at the alignment level in order to reduce noise. Currently three multiple template combination methods are implemented. The first is the *structure-alignment-guided, central-star, top-down approach* combination method to integrate every list of query-template alignments directly generated by each search tool. The method first selects a top ranked query-template as a seed. Using the common query sequence as an anchor, it combines other template-query alignments ranked lower in the list with the seed if their e-values are close to the seed alignment and their aligned regions are structurally consistent with previously combined query-template alignments. The structural similarity of two query-template alignments is checked by comparing the structure of two templates which align to the same regions of the query (as determined by TM-align [24]). Two regions that could be structurally aligned with a high structural similarity score (i.e. GDT-TS score [25] > 0.75) are considered to be structurally conistent. The *structural consistency check* ensures the structural consistency of combined templates and improved model quality by avoiding or reducing atom clashes that result from the combination of structurally inconsistent templates. The second approach called “*structure-alignment-driven profile alignment*” is applied to the consensus list of templates that do not include query-template alignment information. The method can also generate structurally consistent alignments between a query and multiple templates. For each template in the list, the method first aligns its structure with that of each of the remaining templates using TM-align [24]. Each pairwise template-template structure alignment is converted into a pairwise sequence alignment by retaining only structurally aligned residues in the template. These pairwise sequence alignments between the common template and other templates in the list are combined into a multiple sequence alignment using the common template as an anchor. Because only those regions of the other templates that aligned well to the anchor template are kept, the multiple sequence alignment involving multiple templates is *structurally* consistent. The multiple sequence alignment (resp. HHSearch [14] profile) of these templates is then aligned with the multiple sequence alignment (resp. HHSearch profile) of the query to generate an alignment between the query and all the templates using the multiple sequence alignment tool MUSCLE (resp. HHSearch). The third approach is a *hybrid alignment combination* approach that gradually combines the alignments of a query-template pair generated by three different alignment methods: PSI-BLAST [13], HHSearch [14], and SPEM [26]. More specifically, this approach works by taking the PSI-BLAST alignment method first and then adding the HHSearch alignment for query regions not covered by PSI-BLAST alignment if available. Finally the SPEM global alignments are included for the rest of the uncovered query regions if available. The hybrid approach tries to supplement the shorter, but likely more confident local alignments (e.g. PSI-BLAST) with longer, but perhaps less accurate global alignments (e.g. SPEM). Through the second and third methods, a list of combined query-template alignments is generated for the consensus template list. The two structure-alignment guided template combination methods that ensure the structural consistency among multiple templates and the hybrid combination method are new developments in the MULTICOM system.

#### Model generation

Each combined query-template alignment and the associated template structures are fed into model generators to sample conformations for the query protein. If one or more templates are found to cover the entire query protein, leaving no unaligned region or very short unaligned regions (< 10 residues), then the template-based modeling tool (Modeller 9v7 [27]) is used to generate a number of conformations (e.g. 10) for one set of input alignment and template structures. The model best fitting the restraints extracted from template structures is selected as the output model for the set of inputs. As such, a list of models will be generated for the list of input alignments and template structures. About 30-40% of the time, no homologous templates or only a template covering a part of the query protein is found, so a recursive protein modeling protocol [28] is used to integrate template-based modeling method and template-free modeling method to construct conformations that cover the entire query protein. Under this protocol, the certain regions of the query that align well with templates are first constructed by a comparative modeling tool - Modeller [27]. While keeping the conformations of template-based regions fixed and as restraints, a variant of a fragment-assembly tool (i.e. Rosetta [29]) is used to sample the conformations for the uncertain/unaligned regions. This method took the internal core region modeled by template-based modeling into consideration when calculating the energy while keeping the core rigid. This approach can integrate template-based and template-free modeling at a percentage from 0% to 100% depending on the amount of template information available. The conformations of certain and uncertain regions are then composed into a full model using Modeller. In the end, the model generation layer will produce a pool of candidate models (e.g. a few hundred) for the query protein. In this layer, the method of combining template-based and template-free models is a new addition.

#### Model quality assessment

The model quality assessment layer evaluates the quality of each model in the pool in order to select more accurate models. There are two kinds of model quality assessment (or model selection) methods, which can be referred to as the *white box* approach and the *black box* approach. The white box approach uses the information applied in generating a model to evaluate its quality. A typical method of the white box approach is an alignment-based model selection method [30,31] which uses the level of the similarity between query-template alignments (e.g. e-value of alignment score, sequence identity) to rank models generated from the alignments. The method of the black box approach uses the features extracted from the 3D shape of a model to assess its quality without exploiting any specific information about how the model is generated. In contrast to the scarcity of the white box methods, a variety of the black box model selection methods (e.g., energy-based methods [32-34], machine learning methods [35-38], and consensus methods [39-42]) have been developed. Note that white box methods are not always applicable since the information related to how a model is generated is often not available. However, if there is such information, the white box approach tends to provide new insights into the quality of a model that might not be captured by the black-box methods.

Because there is no white-box model quality assessment method publicly available, we developed a support vector machine (SVM [43]) method to predict the quality score of a model based on the features extracted from the query-template pairwise sequence alignment employed to generate the model. The input features provided to the SVM predictor include the logarithm of e-value of the given query-template alignment, the percent of identical residue pairs in aligned positions, the percent of residues of the query that are aligned with a residue in the template, and the average of BLOSUM scores of all aligned residue pairs. From the input feature of a query-template alignment, the SVM predictor aims to predict the GDT-TS score of the model generated from the alignment. The input feature vectors in the training data set were extracted from 245 pairwise protein sequence alignments generated for 50 CASP9 targets by PSI-BLAST [13] and the output score of each input feature vector was the real GDT-TS score of its corresponding model calculated by the TM-score program [44]. This data was used to train a SVM regression predictor equipped with a Gaussian radial basis kernel (RBF) to predict the GDT-TS scores of models from the input features. The three parameters of the Gaussian radial basis kernel (RBF) to be tuned were the epsilon width of the regression tube (w), the margin-error tradeoff parameter (c), and the gamma of the RBF kernel (g). The root mean square error (RMSE) and the absolute mean error (ABS) between predicted and real GDT-TS scores were calculated for each set of parameter values to evaluate its performance. A five-fold leave-one-out cross validation (LOOCV) protocol was used to select the best parameter values of *c* from 2.0, 1.0, 0.5, 0.1, 0.05, 0.01, w from 0.5, 0.2, 0.1, 0.05, 0.02, and 0.01, and g from 0.5, 0.3, 0.2, 0.1, 0.05, 0.01, 0.005, and 0.001 according to the ABS and RMSE on all the five folds. The global average RMSE and ABS of the SVM trained with the best parameter values on the five-fold training data set were 0.083 and 0.061, respectively. The trained SVM predictor was applied to predict the GDT-TS scores of models of 46 CASP9 targets not used in training from the input features extracted from the corresponding PSI-BLAST alignments.

As model assessment is very challenging and none of the current methods can consistently select the best model, three model quality assessment methods (single-model approach, model pairwise comparison approach (APOLLO) [45], and a hybrid approach [9,46]) are employed to assess the quality of the models in this layer. The single-model method (i.e. ModelEvaluator [35]) assigns an absolute quality score (e.g. GDT-TS score, the expected similarity between the model and the native structure) to each model by comparing the secondary structure, solvent accessibility, contact map, and beta-sheet topology of the model with that predicted from the query sequence [19,47,48]. This method is generally effective at discriminating good models from poor models. The pairwise comparison method (APOLLO) compares a model against all other models using a structure alignment tool (e.g. TM-score [44]) and calculates their similarity in terms of GDT-TS score, TM-score, and MaxSub score. The average similarity between a model and all other models is used as the predicted quality of the model. Note that the accuracy of the pairwise comparison method is input dependent (i.e. it works well only if the size of the model pool is large enough and the largest group of similar models in the pool are of good quality). The hybrid method is a compromise between the single-model method and the pairwise-comparison method. It first ranks the models by the quality scores predicted by ModelEvaluator. The top several (e.g. 5) models are selected as reference models, against which each model is compared. The average similarity between a model and the reference models is used as the quality score of the model. Furthermore, the average distance between a residue in a model and its counterpart in the reference models is used as the local quality of the residue (i.e. its deviation from the native structure). In addition to the three methods above, three simple scoring metrics were also tested. These additional methods included secondary structure scoring, secondary structure segment scoring, and solvent accessibility scoring. The secondary structure ranking method uses the percent of the secondary structures predicted from the sequence of a target that agree with those extracted from a model of the target to rank models.. The idea of secondary structure segment score ranking is similar to the secondary structure ranking except the percent of agreement between secondary structure segments rather than between secondary structures of individual residues is used. Similarly, the solvent accessibility score ranking method uses the percent of the solvent accessibilities predicted from the sequence of a target that agree with those extracted from a model of the target to rank models. With all three simple scoring metrics, a higher score corresponded to a higher model ranking. At the end of this layer, all models in the pool have been ranked by the quality scores predicted by these three scores. In this layer, the alignment-based model evaluation and the pairwise model evaluation are new developments in the system.

#### Model refinement

This last layer of the system uses a top-down local–global model combination approach to combine the top ranked models with other models that were globally very similar to it (e.g., pairwise GDT-TS score > 0.7) or combines very similar local regions of other models if no globally similar models were found. The model combination is essentially a model averaging process which in many cases can produce a model better than the top ranked model or even the best model in the pool. In addition to model combination, some regions of models are also refined according to the local quality. The poorly predicted local regions (e.g. tail regions) are resampled by a modified fragment-assembly method (a Rosetta variant), which keeps the other regions fixed and uses them as restraints to constrain the free modeling of the local regions. However, since some poorly predicted local regions are actually disordered regions, refinement on these regions cannot improve the global quality of the model. Finally the top refined models are released from the system as the final predictions.

According to the description of the five steps above, many database search/alignment tools are used in the MULTICOM protein structure prediction system. BLAST [13,21] (Basic Local Alignment Search Tool) is a tool for finding local similarity between sequences. PSI-BLAST [13] (Position-Specific Iterative Basic Local Alignment Search Tool) is a tool for detecting distant relationships between proteins. COMPASS [15] is a tool for comparison of multiple protein alignments with assessment of statistical significance. HHSearch (version 1.2 and 1.5) [14] is a tool for detecting remote homologues of proteins and generating high quality alignments for homology modeling and function prediction. HMMER [18] is a tool for searching sequence databases for homologs of protein sequences and for finding protein sequence alignments using probabilistic models (profile HMMs). PRC [16] is a stand-alone tool for aligning and scoring two profile hidden Markov models. CS-BLAST [22] is an extension to standard NCBI BLAST that allows an increase in sensitivity by a factor of more than two at the same speed. CSI-BLAST [22] is an extension of CS-BLAST for iterative search with position-specific scoring matrices, two search iterations of which are more sensitive than five search iterations of PSI-BLAST. PSI-BLAST-multi is a top-down PSI-BLAST alignment combination approach to protein structure prediction and its assessments. SAM [17] (Sequence Alignment and Modeling system) is a profile HMM and sequence alignment tool. The alignments of all these tools except for BLAST and PSI-BLAST were combined into one-query and multiple-template alignment by the *structure-alignment-guided, central-star, top-down approach* for model generation. Individual BLAST and PSI-BLAST alignments were used for model generation. The consensus templates found by these alignment tools were used to generate query-template alignments by the *structure-alignment-driven profile alignment* approach. CENTER stands for one-query and multiple-template alignment by MUSCLE, while STAR stands for one-query and multiple-template alignment by HHSearch. CONSTRUCT denotes the hybrid query-template alignment derived from the PSI-BLAST, HHSearch and SPEM. The performance of these individual methods and their combination are discussed in the results and discussions section.

## Results and discussions

### System testing, integration, and environment

As shown above, a sophisticated protein structure prediction system can be rather complicated and many choices and decisions must be made in each layer of the system. Thus integrating the components into one system that performs better than the simple sum of all the components is as critical as assembling computer components into a high-performance computer system. In order to objectively measure the performance of our integrated system, we blindly tested it in the 9^th^ Critical Assessment of Techniques for Protein Structure Prediction (CASP9, http://predictioncenter.org/casp9/) in 2010. CASP9 released 129 protein targets whose structures were not available to the community. After some of the targets were canceled due to prematurely leaked information or difficulties in experimentally determining the structure, 107 official targets are available to assess the performance of the system. The set is sufficiently large and contained diverse types of protein topologies at different levels of difficulty, making it an ideal dataset to objectively benchmark the MULTICOM system. Four variants of the MULTICOM system participated in the CASP9 as four automated server predictors: MULTICOM-CLUSTER, MULTICOM-REFINE, MULTICOM-NOVEL, and MULTICOM-CONSTRUCT. The MULTICOM servers generated a large amount of intermediate data in each step of predictions. The raw data was analyzed in this work to study and compare the performance of the components of each layer during the CASP9 experiment. The analysis provided useful information for tuning the parameters of the components and the entire system.

The entire MULTICOM system was installed and run on a workstation with 8 cores, 8 G of memory and a 1 TB hard disk during the CASP9 experiment. Essentially, the system can be installed and run on a modern PC. Generally, the system can make predictions for a query protein within a timeframe ranging from half an hour to several hours, depending on the length and the difficulty of the target. Prediction times for average-length template-based targets are shorter than average-length template-free targets because template-based targets do not require invoking the more time-consuming template-free modeling tools.

In order to investigate its design and performance, we evaluated the first four steps of the MULTICOM protein structure prediction system by comparing the templates, alignments, and models generated by all kinds of database search/alignment tools, comparing different model generation methods and comparing different model quality assessment tools.

### Comparison of template identification methods

**Figure 2:**
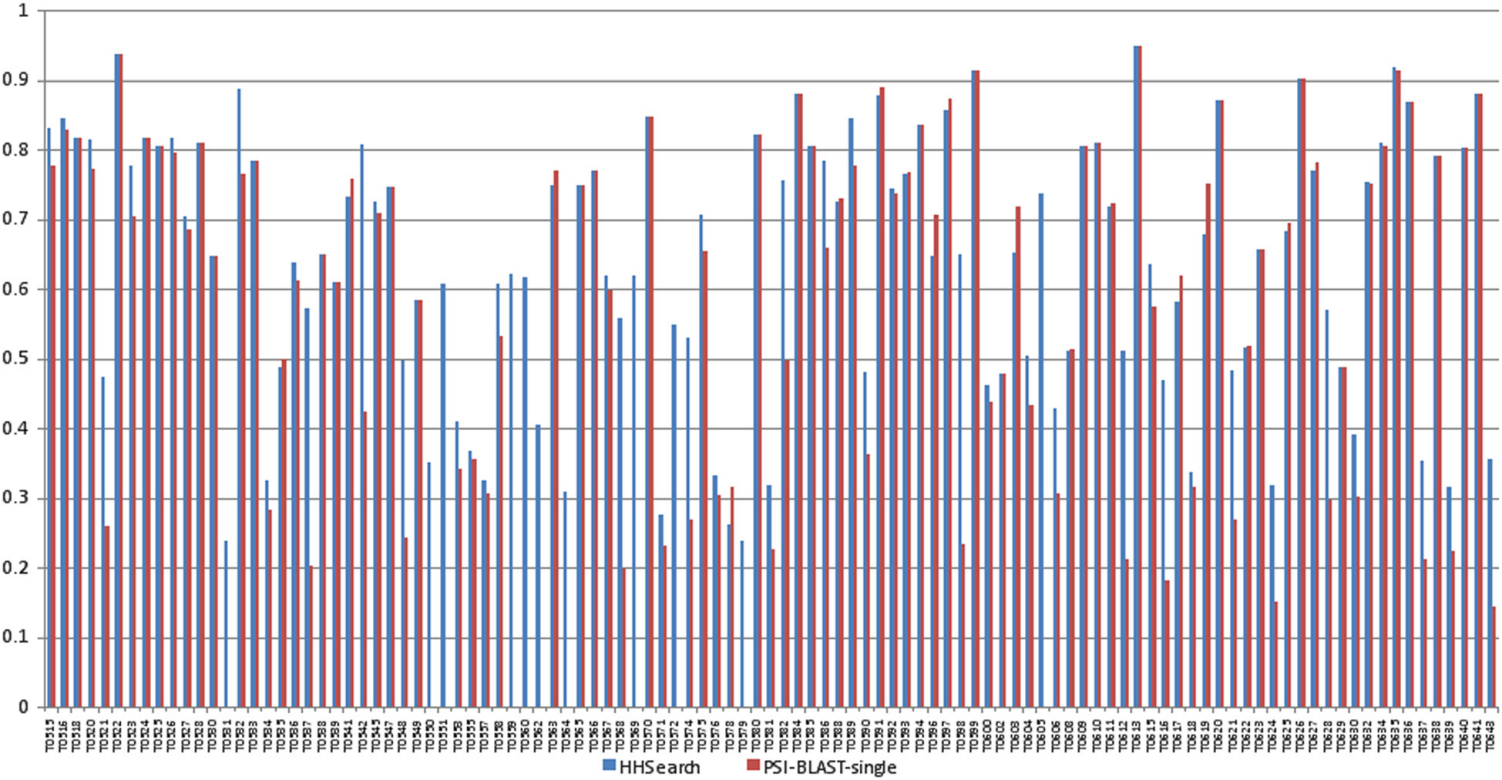
**The highest TM-scores of the top 5 templates searched by HHSearch and PSI-BLAST-single for 107 CASP9 targets. **Y axis represents TM-scores. X axis denotes the index of each target.

In order to evaluate all database search/alignment tools in the first step (i.e., template identification) we compared these tools from different aspects based on the templates identified by each of them. Firstly, the top 5 templates identified by two database search/alignment tools HHSearch [14] and PSI-BLAST-single for 107 CASP9 targets were aligned with the query’s true structure, and their TM-scores were calculated using the TM-align program [24] in order to assess the performance of these two tools in template identification. TM-score [44] is a score in the range of 0 to 1, measuring the similarity between two protein structures and is largely independent of protein length. Here, HHSearch and PSI-BLAST were compared because they are two typical profile-profile and profile-sequence alignment methods. Figure 2 illustrates the highest TM-scores of the top 5 templates identified by HHSearch and PSI-BLAST-single for 107 targets. HHSearch and PSI-BLAST-single identified the templates of the same quality for 25 targets. HHSearch obtained better templates for 60 targets, while PSI-BLAST-single recognized 22 better templates. It is consistent with previous observations that profile-profile alignment methods are more sensitive in recognizing templates than profile-sequence alignment methods. However, profile-sequence alignment can complement profile-profile alignment methods by identifying better templates in some cases.

**Figure 3:**
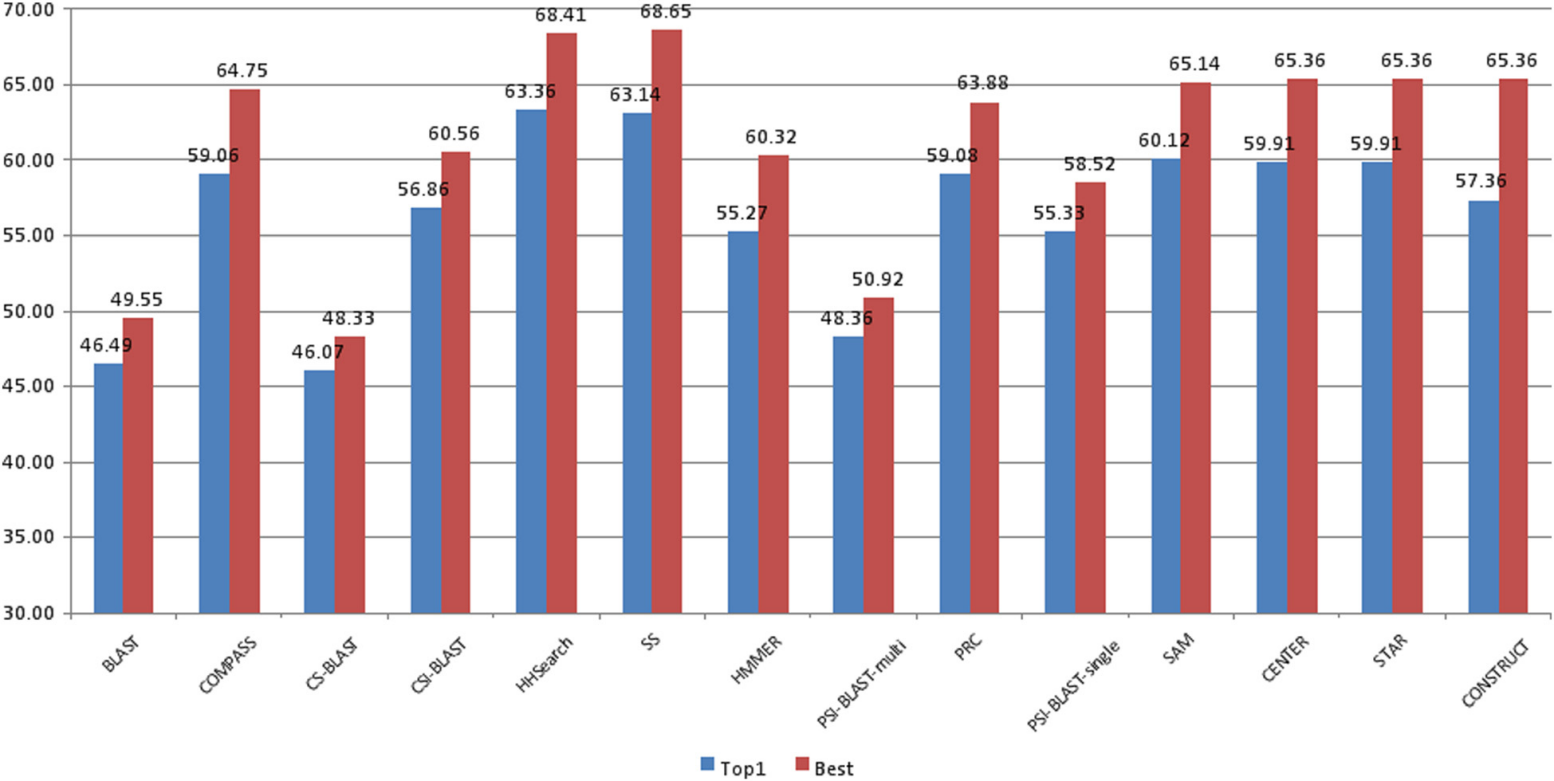
**The total TM-scores of the top 1 template and the best template of each tool for 107 CASP9 targets. **HHSearch is HHSearch version 1.2 and SS is HHSearch version1.5. PSI-BLAST-multi is the multi-template combination of the PSI-BLAST alignment, which had higher total GDT-TS score than the single-template PSI-BLAST alignment approach. Here, the total TM-Score of the top-one templates is the sum of the TM-Scores of the no. 1 template identified for 107 CASP9 targets by a method / tool. Similarly, the total TM-Score of the best templates is the sum of the TM-Scores of the best template identified for 107 CASP9 targets by a method / tool.

**Figure 4:**
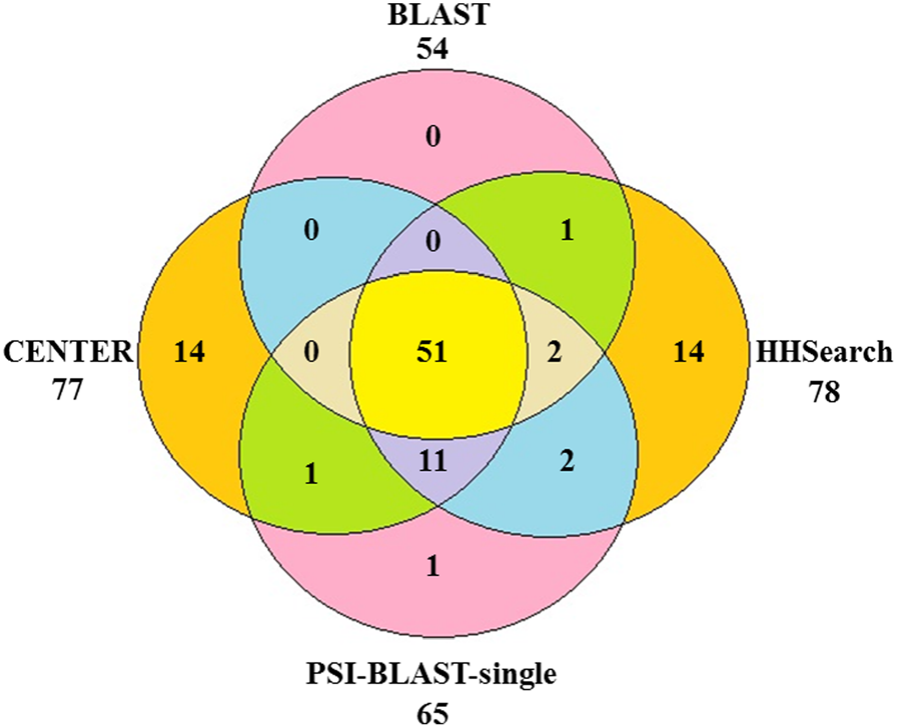
The common and different sub-set of targets for which some good templates (TM-score > 0.5) were identified.

Then we evaluated all of the tools from another aspect by aligning the top 5 templates selected with the query’s true structure for 107 CASP9 targets. Their similarities (i.e. TM-scores) were calculated using the TM-align program [24]. CONSTRUCT is a consensus template identification method that ranks templates based on the frequency of their selection by the other methods. PSI-BLAST-multi used PSI-BLAST to search a query against the NR database to build a PSSM profile and then searched the profile against the template library to select template structures. One difference between PSI-BLAST-multi and PSI-BLAST-single is that the latter searched the NR database for more iterations to include more remote homologous sequences into profile building. Another difference is that PSI-BLAST-multi combined the alignments between one query and multiple templates while PSI-BLAST-single only used one query-template alignment for model building. Figure 3 illustrates the total TM-scores (the addition of all TM-scores) of the top 1 template and the best template with the highest TM-score among the top 5 templates for each tool for 107 CASP9 targets. In both cases, two HHSearch-based profile-profile alignment methods (HHSearch and SS) delivered the best results, followed by the consensus methods (Center, Star, and SAM). Figure 4 illustrates the common and different sub-set of targets for which some good templates (TM-score > 0.5) were identified when using HHSearch, CENTER, BLAST, and PSI-BLAST-single and demonstrated that these methods might identify a complementary set of templates.

Table 1 shows the specificity and sensitivity for the top 1 template and the best template among the top 5 templates for each tool and the number of targets that have templates identified for each tool. It shows that HHSearch, SS, CONSTRUCT, CENTER, and STAR found at least one template for each target of 107 targets. The templates found for around two thirds of the targets were good (TM-score > 0.5). Although it only identified templates for 71 targets, PSI-BLAST-multi got the best specificity for the top 1 model and the best model, which means that the templates searched by PSI-BLAST-multi for more than 80% targets were good templates (TM-score > 0.5) (see Table 1).

**Table 1:** The specificity and sensitivity for the top 1 template and the best template among the top 5 templates for each tool based on 107 CASP9 targets and the number of targets that have templates identified for each tool

**Tool**	**The top 1 model**		**The best model**		**# targets that have templates identified**
	**Specificity**	**Sensitivity**	**Specificity**	**Sensitivity**	
PSI-BLAST-multi	80.28%	53.27%	88.73%	58.88%	71
CS-BLAST	73.97%	50.47%	78.08%	53.27%	73
CENTER	67.29%	67.29%	71.96%	71.96%	107
STAR	67.29%	67.29%	71.96%	71.96%	107
HMMER	66.67%	56.07%	77.78%	65.42%	90
SS	66.04%	65.42%	71.96%	71.96%	107
HHSearch	65.42%	65.42%	72.90%	72.90%	107
BLAST	65.38%	47.66%	69.23%	50.47%	78
CSI-BLAST	62.63%	57.94%	66.67%	61.68%	99
COMPASS	62.50%	60.75%	71.15%	69.16%	104
PSI-BLAST-single	62.50%	56.07%	67.71%	60.75%	96
PRC	62.14%	59.81%	69.90%	67.29%	103
SAM	61.32%	60.75%	67.92%	67.29%	106
CONSTRUCT	60.75%	60.75%	71.96%	71.96%	107

The specificity is the fraction of the targets with at least one template identified by a method having a GDT-TS score > = 0.5, i.e. the number of targets for which a good template is identified divided by the number of targets for which at least one template is identified. The specificity measures the precision of template identification of a method. The sensitivity is the number of targets for which a good template (i.e. its GDT-TS score >0.5) is identified divided by all the targets in consideration in this experiment (i.e. 107), assuming that all the targets have at least one reasonable template. The two measures (i.e. sensitivity and specificity) are complementary.

### Impact of alternative templates and alignments, alternative methods, structural consistency checking, and multiple-template combination on model accuracy

**Figure 5:**
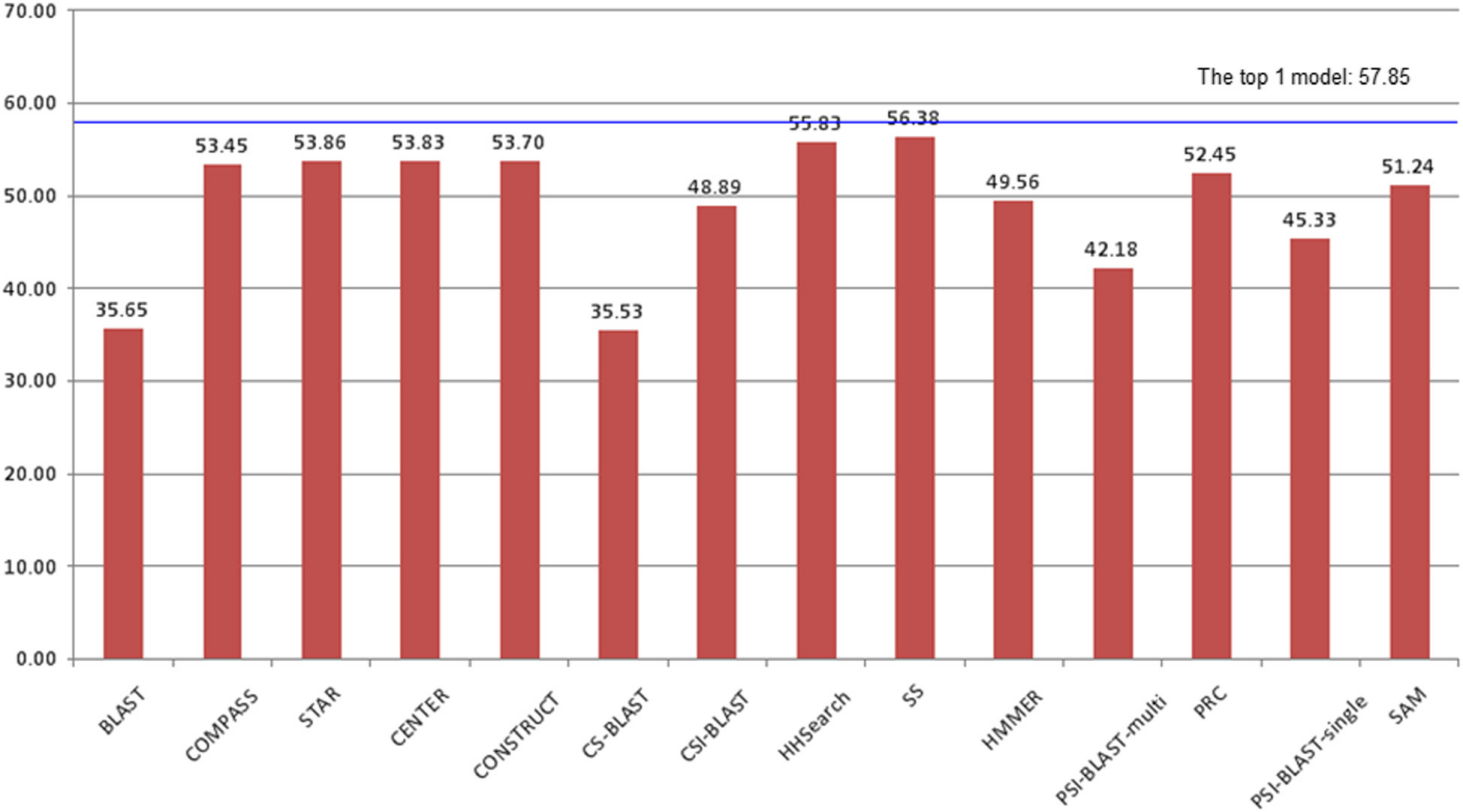
**The total GDT-TS scores of the top 1 ranked model of each individual method and the top 1 ranked models of all of the methods for 107 CASP9 targets. **The vertical bars represent the total scores of individual methods. The blue line denotes the total score of top 1 model of all the methods.

**Figure 6:**
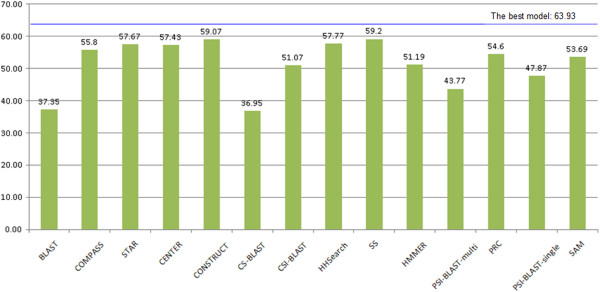
**The total GDT-TS scores of the best model of each individual method and the best model of all the methods for 107 CASP9 targets. **The vertical bars represent the total scores of individual methods. The blue line denotes the total score of the best model of all the methods.

In order to explore the impact of multiple-template combination of all of the tools (BLAST [13,21], CS-BLAST [22], CSI-BLAST [22], HHSearch [14] with different profiles, PRC [16], COMPASS [15], HMMER [18], SAM [17], PSI-BLAST-single, PSI-BLAST-multi, CONSTRUCT, CENTER, and STAR), the top 5 models generated by these tools for 107 CASP9 targets were superimposed onto the query’s true structure and the GDT-TS scores were calculated by the TM-score program [44]. GDT-TS (Global Distance Test) score is the average percent of residues in the model whose position is within 1, 2, 4, 8 Angstrom with that of their counterparts in the experiment structure after superposition [25]. Figure 5 reports the total GDT-TS scores of the top 1 models of each individual method and the total GDT-TS score of the top 1 models among all the models of all the methods. Figure 6 reports the total GDT-TS scores of the best models with highest GDT-TS score of each individual method and the total GDT-TS score of the best model with the highest GDT-TS score among all models of all the methods. As shown in Figures 5 and 6, the score of HHSearch 1.5 (i.e. SS) on a filterd profile is slightly higher than that of the other tools, which reveals this method generated better target-template alignments. However, the total score of the method was still a few percent lower than the total score of top ranked or the best models generated from the target-template alignments of all the methods. This suggests that pooling models generated from alternative target-template alignments produced by the different methods improved model quality.

Table 2 shows the total GDT-TS scores of PSI-BLAST-multi and PSI-BLAST-single for the top 1 model and the best model on the same set of 71 targets for which both methods made predictions. The results show that PSI-BLAST-multi has a slightly better performance than PSI-BLAST-single. However, it was hard to quantify the contributions of multiple template combination here because the templates used for each target by the two methods may be different.

**Table 2:** The total GDT-TS scores of PSI-BLAST-multi and PSI-BLAST-single on the same set of 71 targets for which both methods made predictions

**Tool**	**Total GDT-TS score**	
	**The top 1 model**	**The best model**
PSI-BLAST-multi	42.18	43.77
PSI-BLAST-single	41.51	43.33

In order to investigate the impact of structural consistency checking for HHSearch modeling, we assessed and compared three kinds of HHSearch [14] models (i.e. HH with structural consistency checking, SS with structural consistency checking, and HS without structural consistency checking). All of the generated models of HH, SS, and HS for 107 CASP9 targets were aligned with the query’s true structure, and their GDT-TS scores were calculated using the TM-score program [44]. The total GDT-TS scores of the best models of HH and SS with structural consistency checking are 57.77 and 59.2 respectively, clearly higher than that of HS without the consistency check which scores 52.44. In spite of some difference in HHSearch versions, profiles, and other parameters, this may still imply that methods with structural consistency checking have better performance than methods without a structural consistency check.

STAR models (HMM), CENTER models (MUSCLE), and CONSTRUCT models were compared in order to assess the quality of the multiple sequence alignments generated. All of the generated models of STAR, CENTER, and CONSTRUCT for 107 CASP9 targets were aligned with the query’s true structure and their GDT-TS scores were calculated using the TM-score program [44]. The total GDT-TS scores of the best models of STAR, CENTER, and CONSTRUCT with highest GDT-TS score for 107 CASP9 targets are 57.67, 57.43, and 59.07 respectively (see Figure 6), whereas the total GDT-TS scores of the top 1 ranked models of these methods are similar (see Figure 5).

### Comparison of model generation protocols

We compared the performance of the *ab initio* model generation method and the template-based method on hard targets by comparing HHSearch models, SS models and *ab initio* models. Hard targets are template-free targets that did not have a reasonable template in the protein structure database. All of the generated models of HHSearch, SS, and *ab initio* for 8 CASP9 hard targets [49] were aligned with the query’s true structure and their GDT-TS scores were calculated using the TM-score program [44]. The total GDT-TS score of the best models of *ab initio* with the highest GDT-TS score is 2.55, clearly higher than 1.88 of HHSearch and 1.79 of SS. This suggests that the *ab initio* models generated by the fragment assembly based *ab initio* method were better than the models generated by the template-based method with incorrect templates.

We further compared four template-based model generation protocols (i.e. auto model, loop model, dope_loop model, and dope_hr_loop model) of Modeller [27]. All of the models generated by these four protocols using HHSearch [14] alignments for 107 CASP9 targets were aligned with the query’s true structures. Their GDT-TS scores were calculated using the TM-score program [44]. Table 3 illustrates the total GDT-TS scores of the best models with highest GDT-TS score generated by these protocols. It was quite surprising that the total GDT-TS score of the simplest auto model protocol is clearly higher than the other, more advanced protocols.

**Table 3:** The total GDT-TS scores of the best models generated by four model generation protocols for 107 CASP9 targets

**Method**	**The total GDT-TS score**
auto model	53.55
loop model	48.41
dope_loop model	47.95
dope_hr_loop model	48.04

### Comparison of model selection methods

We evaluated two kinds of model quality assessment methods (the *white box* approach and the *black box* approach) on the CASP9 targets. We applied the SVM alignment-based predictor (the white box approach) trained on alignments of 50 CASP9 targets to blindly score the models generated from 225 PSI-BLAST-single alignments on the other 46 CASP9 targets. The total real GDT-TS score of the top 1 models selected by the SVM predictor for these targets was compared with that of the top 1 models simply ranked by the e-values of the PSI-BLAST alignment. The total GDT-TS score of the models selected by the SVM predictor is 20.95, higher than 20.10 of the naïve e-value based model selection method. Moreover, a t-test and a wilcox-test were performed to check if the two scores are significantly different (p-value < 0.05). The p-value of t-test is 0.044 and the p-value of wilcox-test 0.042. The results indicate that incorporating multiple alignment features in a SVM can significantly improve model selection over a naïve e-value based method.

**Figure 7:**
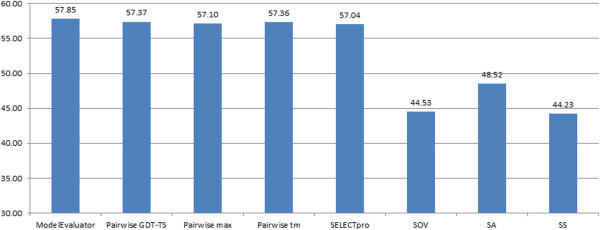
The total GDT-TS scores of the top models selected by different model-ranking technologies for 107 CASP9 targets.

As for the black box model selection methods, we evaluated a single-model absolute model quality predictor (ModelEvaluator), the secondary structure score ranking method, the solvent accessibility score ranking method, the secondary structure segment (SOV) score ranking, a pairwise model comparison method (APOLLO), and an energy ranking method (SELECTpro [32]). APOLLO generated three kinds of scores for a model, i.e. TM-score, GDT-TS score, and Max-Sub score, and these were evaluated separately. All these methods were used to select one model with the highest predicted score from all the models predicted for each of the CASP9 targets. The total real GDT-TS scores of the models selected by each method is reported in Figure 7. The results show that ModelEvaluator yielded the best performance, which is only slightly better than that of SELECTpro and APOLLO. The performance of these three comprehensive quality predictors was substantially better than that of the ranking based methods on a single feature (i.e., SS, SA, SOV).

In addition to evaluating the quality of a model based on the coordinates of all of its residues, we investigated if removing potentially disordered regions from full-length models could improve model quality assessment. In contrast to previous work that excluded potentially disordered residues from model generation resulting in a partially constructed model, our approach removes them from a full-length model containing all the residues in order to improve the accuracy of evaluating its quality. We used PreDisorder [50] to predict the putative disordered residues of each target and then filtered out the coordinates of the N-/C-terminal disordered residues from all the models. ModelEvaluator, APOLLO, and SELECTpro were used to assess the filtered models and to select one model with the highest score from all the filtered models for each of the CASP9 targets. The performance of these methods applied to the filtered models was compared with that of the same methods when applied to the full-length models. The total real GDT-TS scores of the best models selected by these methods are reported in Table 4. The results show that removing N/C-terminal disordered regions from full-length models improves the performance of all the quality assessment methods. The improvement on the pairwise quality assessment method (Apollo) and the energy-based method (SELECTpro) was more pronounced, indicating that these methods were more sensitive to the noise caused by the disordered residues than ModelEvaluator. Overall, our experiment suggests that disorder prediction may help significantly improve model ranking, which has been a long-standing and challenging problem.

**Table 4:** The total GDT-TS scores of the best models without the tail disorder regions and the best models with the tail disorder regions for 107 CASP9 targets

**Model**	**The total GDT-TS score**				
	**ModelEvaluator**	**APOLLO tm**	**APOLLO max**	**APOLLO GDT-TS**	**SELECTpro**
The best model without the tail disorder regions	57.88	61.12	60.92	61.01	59.94
The best model with the tail disorder regions	57.85	57.36	57.10	57.37	57.04

### Impact of model combination and refinement on model quality

In order to assess the impact of the simple model combination and refinement method on model quality, we compared the total GDT-TS score, TM-Score and MolProbity score of the combined models with those of the top ranked models of 107 CASP9 targets (see Table 5). MolProbity differs from the GDT-TS and TM-Score metrics in that MolProbity evaluates how realistic a model is according to its all-atom conformation. GDT-TS and TM-Score measure the accuracy of the backbone of a model. The results show that the GDT-TS scores and TM-Scores of the combined and refined models and the top ranked models are almost the same while the MolProbity score of the former is better (i.e. lower) than that of the latter. This suggests that combining / refining models may make models more protein-like.

**Table 5:** The total TM-score, GDT-TS score, and MolProbity score of the combined, refined models and the top selected models of 107 CASP9 targets

**Models**	**TM-score**	**GDT-TS score**	**MolProbity score**
The combined, refined models	64.20	57.14	340.98
The top selected models	64.28	57.21	351.18

## Conclusion

Developing high-quality protein structure prediction systems is critical for addressing the protein structure challenges faced in the post genomic era. In this work, we described how to construct a protein structure prediction system (MULTICOM) under a five-layer open architecture, which can integrate complementary component methods and multiple sources of information to reliably and accurately predict protein structure from sequence. We focused on investigating and validating the effectiveness and complementarity of different components employed in each layer. The experiments provided insights about how to select, use, and combine existing techniques to improve protein tertiary structure prediction using an open architecture. Additionally, the experiments provide a direct, comprehensive and quantitative assessment of various components of a single protein structure prediction system in a blind prediction setting and lead to some interesting findings such as the impact of protein disorder prediction on protein model selection. These results shed new light on designing and developing better protein structure prediction systems and algorithms.

However, despite the reasonable performance that the MULTICOM protein structure prediction system achieved on most protein targets, our benchmark suggests there is still room for improvement in each step of protein structure prediction process. In the future, we plan to add more sensitive or complementary template identification methods into the system to address the failure of identifying good templates for some hard targets. These improvements will include more complementary or even better alignment methods to generate more accurate target-template alignments, improve alignment-based model quality assessment methods with more features and multiple-template information, incorporate residue-residue contact information to improve *ab initio* model generation (i.e., a major bottleneck of protein structure prediction), and explore the usage of residue disorder prediction in both template-based and *ab initio* model generation.

## Competing interests

The authors declare that they have no competing interests.

## Authors’ contributions

JC conceived the system. JC, JL, XD, JE designed, developed and tested the system. JC, JL, XD, JE authored, edited and approved the manuscript. All authors read and approved the final manuscript.
